# Effect of Three Defatting Solvents on the Techno-Functional Properties of an Edible Insect (*Gryllus bimaculatus*) Protein Concentrate

**DOI:** 10.3390/molecules26175307

**Published:** 2021-08-31

**Authors:** Min-Soo Jeong, Sang-Deok Lee, Seong-Jun Cho

**Affiliations:** 1 Department of Food Science and Biotechnology, College of Agriculture and Life Sciences, Kangwon National University, Chuncheon 24341, Korea; jminsoo@kangwon.ac.kr; 2 Division of Forest Science, College of Forest and Environmental Sciences, Kangwon National University, Chuncheon 24341, Korea

**Keywords:** insect, cricket, solvent extraction, secondary structure, protein denaturation

## Abstract

Edible insects have received global attention as an alternative protein-rich food. However, their structural characteristics make them difficult to digest. To overcome this obstacle, we assessed the techno-functional properties of three protein concentrates from the cricket *Gryllus bimaculatus*. Freeze-dried *G. bimaculatus* powder was defatted using ethanol, hexene, or acetone as solvents, and the techno-functional properties (protein solubility, water and oil holding capacity, foaming properties, emulsion capacity, and gel formation) of the protein concentrates were determined. Freeze-dried *G. bimaculatus* powder comprised approximately 17.3% crude fat and 51.3% crude protein based on dry weight. Ethanol was the most effective solvent for reducing the fat content (from 17.30% to 0.73%) and increasing the protein content (from 51.3% to 62.5%) of the concentrate. Techno-functionality properties drastically differed according to the defatting solvent used and foaming properties were most affected. Thus, the techno-functional and whole properties must be considered for proper application of edible insects to achieve global food sustainability.

## 1. Introduction

Reports of the Food and Agriculture Organization of the United States warned that the global population is expected to reach 9 billion by 2050, leading to an approximately 70% increase in food requirements from current demands [1]. Specifically, the annual consumption of meats may increase from 40.0 kg/capita in 2013 to 51.5 kg/capita (129%) in 2050 [2]. In addition, the demand for plant-based dietary proteins such as maize, rice, wheat, and soy is expected to increase by approximately 60–110% by 2050 to meet sufficiency of the global population [3]. Moreover, animal-based protein food production is a major cause of environmental problems such as water shortage and global warming. Considering the above, it is an urgent task to find sustainable sources of protein that can serve as effective substitutes for animal-based protein. Edible insects have received global attention as a potential solution to this problem, resulting in a new active field of research in the food industry. More than 2000 species of insects are currently recognized with potential as human food sources, including beetles (*Coleoptera*), caterpillars (larvae of butterflies and moths; *Lepidoptera*), locusts, grasshoppers, and crickets (*Orthoptera*), and bees, wasps, and ants (*Hymenoptera*). Edible insects contain between 30% and 65% (dry basis) protein, which is higher than that of beans (23.5%), lentils (26.7%), or soybean (41.1%) [4]. Moreover, insects are also a good source of minerals [5].

The two-spotted cricket *Gryllus bimaculatus* belongs to the order *Orthoptera*, which is known to have the highest mean protein content among all insect taxa [6]. Further, *G. bimaculatus* is enriched in other important nutrients such as minerals and vitamins [4]. Accordingly, studies have focused on the nutritional and functional properties of *G. bimaculatus* to harness its potential as an alternative protein source for the human diet [4,7,8]. However, their hard exoskeletons and unpalatable appearance pose the greatest challenge to global acceptance of these crickets as part of the regular diet. In addition to overcoming these issues of consumer preference and acceptance, there are critical difficulties associated with the intake of insects, despite their abundant nutrients [9]. Thus, appropriate processing is necessary to manipulate the shape or form of insect, and much work has been published on the addition of cricket powder to food products, mainly cereals [10,11].

Defatting is one of the most effective processes for increasing the protein content and reducing the lipid content of raw materials. Lipid oxidation is associated with safety and qualitative problems such as the generation of a rancid odor and noxious compounds. Previous studies on defatting processes suggested that solvents with different polarities can impact defatting properties such as the defatting efficiency from krill meal and microalgae and the techno-functional properties of fenugreek protein isolate from fenugreeks defatted by different solvents [12,13,14].

Based on this background, we hypothesized that a defatting process could be an effective method to change the constituents of *G. bimaculatus*, especially lipids, and ultimately improve the techno-functionalities. For effective defatting of insects, the choice of extraction solvent should be considered carefully. Therefore, the aims of this study were to evaluate and compare the techno-functional properties and compositions of three defatted protein concentrates from *G. bimaculatus* powder using three organic solvents (ethanol, hexene, or acetone) that are commonly applied in food processes and have different degrees of polarity. The freeze-dried and defatted powders were then subjected to proximate analysis, along with tests for protein solubility, protein surface hydrophobicity, and assessment of six techno-functional properties.

## 2. Material and Methods

### 2.1. Raw Material and Preparation of Insect Powder

Live *G. bimaculatus* were purchased from a cricket farm (Wonju Natural Ecology Park, Wonju, Korea). The crickets were fed with corn and cabbage and harvested 42 days after hatching. The crickets were fasted for 24 h and then killed by CO_2_ treatment and deep-freezing at −80 °C. The frozen crickets were then lyophilized and ground by a pulverizer (RT-N08, Rong Tsong Precision Technology, Taichung, Taiwan), and the lyophilized cricket powder was maintained at −20 °C until use in the experiments.

### 2.2. Defatting

The insect powder was defatted according to the method described by Choi et al. (2017) [15]. In brief, 100 g of the insect powder were respectively dispersed in 500 mL of each solvent (99.5% ethanol, 97% 1-hexene, or 99.5% acetone; Sigma-Aldrich, St. Louis, MO, USA) with stirring at 25 °C for 2 h. After the first extraction, the mixtures were separated by a filtering device (Whatman No. 4, Whatman PLC, Maidstone, UK) under vacuum. Collected residues were resuspended and filtered using the same procedure with 500 mL of each solvent. Thereafter, the defatted insect powders were left to dry overnight under a fume hood.

### 2.3. Proximate Analysis

All samples were analyzed according to Association of Official Agricultural Chemists (AOAC) methods [16]. The protein content was measured by the Kjeldahl method, in which the digested sample was distilled by an autoanalyzer (Kjeltec8200, Foss, Hillerod, Denmark) using an N conversion factor of 4.76 [17]. The fat content was calculated according to the Soxhlet method by weighing the fat and oil extracted by ether. The ash content was determined by drying the samples in a furnace. The carbohydrate content was calculated according to the following formula:

Carbohydrate content (%) = 100 − Crude protein [%, dry matter basis (DM)] − Crude fat (%, DM) − Crude ash (%, DM) (1)

### 2.4. Protein Solubility

The protein solubility was measured according to the method described by Zielińska et al. (2018) with a slight modification [18]. The samples (0.2 g) were dispersed in 20 mL distilled water and the pH of the 1% suspensions was adjusted from 2.0 to 11.0 by the addition of 0.5 M HCl or 0.5 M NaOH. The protein of the mixture was extracted for 60 min with stirring and then centrifuged at 3200× *g* for 20 min. The protein content in supernatants was measured using the Kjeldahl method, and protein solubility was calculated as the proportion of soluble protein per total protein [16].

### 2.5. Techno-Functional Properties

Water holding capacity (WHC) was determined as described by Zielińska et al. (2018) with a slight modification [18]. The sample (0.8 g) was dispersed in 32 mL of water and stirred at 500 rpm for 30 min. After centrifugation at 3200× *g* for 20 min, the weight of the precipitate was measured. The WHC was then calculated by subtracting the weight of the used sample from that of the precipitate, and expressed as grams of absorbed water versus 1 g of sample in the dry material.

Oil holding capacity (OHC) was also determined as described by Zielińska et al. (2018) with a slight modification [18]. All moisture in the sample was removed using a vacuum-drier before the measurement. The dried sample (0.8 g) was then dispersed in 32 mL of soybean oil and stirred at 500 rpm for 30 min. After centrifugation at 3200× *g* for 20 min, the weight of the precipitate was measured, and the OHC was calculated by subtracting the weight of the used sample from that of the precipitate, expressed as grams of absorbed oil versus 1 g of sample in the dry material.

Foaming properties, including the foam capacity (FC) and foam stability (FS), were determined as described by Antonic et al. (2021) with a slight modification [19]. A 1% suspension in 20 mL distilled water was homogenized using a homogenizer (HG-15A, Daihan) at 16,000 rpm for 2 min. The whipped mixture was then immediately moved into a cylinder, and the foaming properties were calculated using the following formulas:

(2) $$\mathrm{FC}\;(\%)=\frac{V_{0}-V}{V}-100$$

(3) $$\mathrm{FS}\;(\%)=\frac{V_{0}}{V_{30}}\;\times \;100$$

where *V* is the volume of the solution before homogenizing (mL), *V*_0_ is the volume of the solution after homogenizing (mL), and *V*_30_ is the volume of the solution 30 min after homogenizing (mL).

Gelation properties were determined according to the modified method of Lawal et al. (2007) [20]. Each suspension at different concentrations (15–25%) was stirred for 10 min, and an aliquot of the suspension (5 mL) was transferred into a conical tube (15 mL). For gelation of the suspension, the conical tubes were heated in a water bath at 86 ± 1 °C for 30 min and immediately cooled in a beaker with cold water. The conical tubes were then kept in a refrigerator at 4 °C overnight, and the height of the formed gel was measured. When the suspension did not have fluidity, the lowest concentration of the suspension was used as the index of gelation properties.

Emulsion capacity (EC) was determined according to the method of Stone et al. (2013) with a slight modification [21]. Each sample (0.5 g) was dispersed in 50 mL of distilled water. The dispersion was simultaneously stirred using a vertical stirrer and homogenized by homogenizer in a beaker. Soybean oil was continuously added to the dispersion during the stirring and homogenization until the emulsion formed breaks. The total volume of oil added to the suspension versus the weight of the sample (g) was used as the index of EC.

### 2.6. Protein Surface Hydrophobicity

Protein surface hydrophobicity of the samples was measured according to the modified method described by Mishyna et al. (2019) [22]. Each sample was dissolved in 0.2 M phosphate buffer (pH 7.0) and stirred for 1 h. After centrifugation at 3200× *g* for 20 min, the protein concentration in the supernatant was measured by the Bradford method and adjusted to various concentrations (0.005%, 0.01%, and 0.02% *w*/*v*). Fifteen microliters of 8-anilino-1-naphthalenesulfonic acid (0.008 M; Sigma-Aldrich) were added to 3 mL aliquots of each supernatant and left for 15 min in the dark. The fluorescence intensity of the soluble protein was measured by a fluorescence spectrophotometer (LS-55, Perkin Elmer, Waltham, MA, USA) at an excitation wavelength of 390 nm and emission wavelength of 480 nm. The protein surface hydrophobicity was estimated as the slope of the plot of protein concentration vs. fluorescence intensity.

### 2.7. Prediction of Secondary Structure

Changes in the *G. bimaculatus* protein secondary structure after defatting with the different solvents were analyzed using a circular dichroism (CD) spectropolarimeter (J-1500, JASCO, Dunmow, UK). Each sample was diluted to 1 mg/mL in 5 mM potassium phosphate buffer (pH 7.0). The mixtures were centrifuged at 10,000× *g* for 5 min after extraction with stirring for 1 h. The supernatants (200 μL) in a 0.1 cm path-length quartz cell were recorded by the CD spectropolarimeter with a scan rate of 100 nm/min from 190 nm to 260 nm. The scan results were used to estimate the secondary structure of the soluble proteins using the program Spectra Manager 2 (JASCO).

### 2.8. Statistical Analysis

SPSS Statistics (Ver. 24, SPSS, Inc., Chicago, IL, USA) was used for all statistical analyses. The data are presented as the mean ± standard deviation. One-way analysis of variance was conducted to compare data among groups, followed by Duncan’s multiple-range test for pairwise comparisons, with a significance threshold of 5%. Each experiment was performed in triplicate, except for the amino acid analysis that was performed in duplicate.

## 3. Results and Discussion

### 3.1. Proximate Analysis

The proximate components in each defatted *G. bimaculatus* powder are shown in Table 1. Ethanol most effectively reduced the fat content of *G. bimaculatus* from 17.3% to 0.73%, and significantly increased the protein content of the insect from 51.3% to 62.5%. Thus, ethanol, as a polar solvent, was more effective for extracting the oil from *G. bimaculatus* than the non-polar solvents (hexene and acetone). This result is in agreement with a previous report on krill oil extraction, which showed that high phospholipid extraction from krill using polar solvents resulted in a greater lipid extraction yield than obtained when using non-polar solvents [12]. This effect is attributed to the high dielectric constants of polar solvents for extracting polar lipids (phospholipids, glycolipids, and free fatty acids) [23,24]. Thus, use of ethanol as the most effective solvent for polar lipid extraction could result in the lowest fat content of the ethanol-defatted protein concentrate (EDP). Defatting processes using acetone and hexene significantly increased the ash content of freeze-dried *G. bimaculatus* powder (FGP) in the hexene-defatted protein concentrate (HDP) and acetone-defatted protein concentrate (ADP), and decreased that of the EDP. Thus, the crude ash content of *G. bimaculatus* was reduced by approximately 12% and 3% with the ethanol defatting process and with the hexene/acetone defatting processes, respectively.

### 3.2. Protein Solubility

Proteins have techno-functional properties such as WHC and OHC, foaming properties, emulsion capacity (EC), and gel formation ability (GF) in food. Among these properties, the foaming properties, EC, and GF are dependent on the degree of protein solubility. Therefore, to optimize the techno-functional properties of proteins, they must be fully dissolved in an appropriate medium.

**Figure 1:**
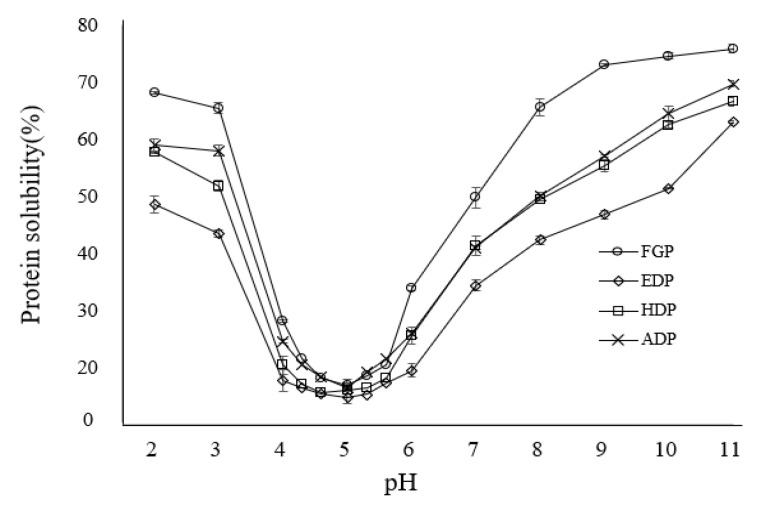
Protein solubility of *G. bimaculatus* and three defatted protein concentrates at different pH values. FGP, freeze-dried *Gryllus bimaculatus* powder; EDP, ethanol-defatted protein concentrate; HDP, hexene-defatted protein concentrate; ADP, acetone-defatted protein concentrate.

Figure 1 shows the protein solubilities of the four samples at various pH values (2.0–11.0). All of the defatting processes by the solvents used in this study decreased the protein solubilities of *G. bimaculatus* powder over the entire pH range tested. In addition, the solubility of all samples increased when changing the pH from the isoelectric point (pH 5.0) to pH 2.0 or 11.0. Among the three solvents, ethanol resulted in greater decreases of the solubility of proteins from *G. bimaculatus* compared to hexene or acetone with lower dielectric constants. The solubility of proteins is also influenced by the structure of their molecules and the ratio of polar to non-polar groups. Effective reduction of lipids by ethanol may have enhanced the denaturation of proteins by easily allowing contact to patches and regions that were blocked by lipids, therefore resulting in further collapse of the protein structure. Hydration of some proteins in organic solvents was reported to decrease with the decline of protein–water hydrogen bonds and with the increase in the number of protein–protein hydrogen bonds [25].

### 3.3. Techno-Functional Properties

Table 2 shows the six techno-functional properties for the three defatted protein concentrates and original FDG powder.

Among the solvents used for defatting, only ethanol defatting increased the WHC of *G. bimaculatus*, with no significant differences in WHC between the other three samples (FGP, HDP, and ADP). Exposure of water-binding regions on the side chain blocked by lipophilic materials may explain the enhanced water absorption properties of defatted samples [26]. The difference in the effects of defatting solvents on WHC is attributed to various aspects of denaturation owing to their different dielectric constants [25]. Thus, the lowest fat content of EDP (0.7%) resulted in a higher WHC than that of the other defatted protein concentrates. Residual fat in the defatted protein concentrate may inhibit binding interactions between water and protein.

EDP also resulted in the highest oil holding capacity (OHC), whereas the ADP showed the lowest OHC value. Therefore, defatting using ethanol, as the most polar solvent of all solvents used to defat the insect, was the most effective method to increase the OHC of *G. bimaculatus* powder. EDP has the lowest crude fat content and the highest crude protein content, so this enhancement in the OHC of EDP is likely due to the number of non-polar side chains of proteins that bind oil [26].

Among the three organic solvents tested, hexene, as a non-polar solvent with the lowest dielectric constant of all solvents used, was the most effective at enhancing the FC and EC of *G. bimaculatus* powder. The foaming properties and EC values after defatting were inversely proportional to the dielectric constant of the defatting solvents used to defat them (Table 2). To achieve fine foaming, a protein must have the ability to rapidly displace to the air–water interface, thereby rearranging and unfolding at the interface. Foam formation and stability are generally dependent on the proteins forming the interfacial film that can maintain air bubbles in the mixture and delay the rate of foam coalescence [27]. However, foaming properties are not only dependent on the protein type but also on other components present, such as carbohydrates. The fact that non-polar solvent treatments increased the emulsion properties of the proteins more than polar solvent treatments is in agreement with previous results reported by Feyzi et al. (2017) for fenugreek protein isolates [14]. In addition, Majzoobi et al. (2012) reported that a decrease in protein solubility would reduce the protein adsorption and protein migration rate at the water–oil interface, thereby decreasing the emulsion properties of proteins [28]. Therefore, the lowest solubility of EDP can explain its lower EC value. Similar to this finding, a previous study showed positive correlations between the emulsion property and protein solubility of gluten [29].

Gel formation abilities were determined as the minimum concentration of samples that can result in firm gel formation. The gel-forming properties of all protein samples were enhanced compared with that of the FDG sample, and ADP formed a gel at the lowest minimum concentration (Table 2).

### 3.4. Changes of Protein Structure

The techno-functionalities of proteins can be changed according to their denaturation resulting from physicochemical treatments such as heat, alterations of amino acid residues, and solvent treatments. Modification of techno-functionality using solvent treatments occurs from denaturation based on the conversions of the number of protein–protein and protein–solvent interactions, positions of hydrophilic and hydrophobic sites on side chains, and protein structures [25]. To determine the degree of change in the protein structure in the three defatted protein concentrates, we measured the protein surface hydrophobicity using a fluorescence spectrophotometer and predicted the secondary structure of the protein with CD spectroscopy. The surface hydrophobicity and secondary structure composition of proteins from the four samples are summarized in Table 3.

Protein denaturation can result in exposure of some hydrophobic regions and groups hidden inside protein molecules to the outside and surroundings. In general, it is expected that a protein with high surface hydrophobicity will have low solubility because of the propensity for aggregation by hydrophobic interactions. However, the ethanol defatting process resulted in intense aggregation of the *G. bimaculatus* proteins and their insolubilization, thus showing the lowest hydrophobicity and solubility values of all solvents tested. Mao et al. (2012) also found a positive correlation between surface hydrophobicity and milk protein solubility, which they attributed to the fact that when protein solubility increases, the hydrophobic patches become accessible to dye molecules, resulting in increased hydrophobicity [30]. Among the three defatted protein concentrates tested in the present study, EDP showed the lowest solubility over the entire pH range (Figure 1). Thus, ethanol is considered the most effective solvent to denature *G. bimaculatus* proteins.

The predicted protein secondary structure can be used as an indicator of the degree of protein denaturation after processing. Protein secondary structures such as α-helix and β-sheet structures mediate numerous interactions between the amino acids, and can be collapsed by exposure to organic solvents as a dielectric material that converts ionic interactions. Only the EDP and ADP showed significant changes in the protein secondary structural composition after defatting. Specifically, ethanol destroyed the α-helix structure, whereas acetone destroyed the β-sheets in *G. bimaculatus* proteins. Collectively, these differences in the denaturation pattern of *G. bimaculatus* proteins according to defatting solvents indicate that solvents with high dielectric constants induce more considerable changes to the protein structure.

## 4. Conclusions

To provide a reference for the selection of defatting solvents for *G. bimaculatus* toward its application as an alternative sustainable protein source, FDG powder was defatted by three organic solvents (ethanol, hexene, or acetone). Proximate analysis and determination of the techno-functional properties of the three defatted protein concentrates and the freeze-dried powder were conducted to compare the resulting protein quality, and the surface hydrophobicity and secondary structure composition of the protein concentrates were determined to investigate their denaturation trends.

Despite its polarity, ethanol was the most effective solvent for reducing the fat content (0.73%) and increasing the protein content (62.5%) of *G. bimaculatus* powder. Defatting by all three solvents decreased the protein solubilities of *G. bimaculatus* powder over a wide pH range. Among the three defatted protein concentrates, EDP showed the greatest reduction in solubility compared with *G. bimaculatus* powder, indicating that ethanol results in the most intense denaturation on *G. bimaculatus* proteins owing to aggregation between the proteins.

Regardless of solubility and denaturation, the three defatted protein concentrates showed different advantages with respect to techno-functional properties, thus supporting our hypothesis that defatting is an effective method to improve protein properties for application as food ingredients and improved palatability or nutrition. EDP showed good water and oil holding capacities, indicating its potential as a food additive for meats, sausages, breads, and cakes to enhance the chewiness of the foods. By contrast, HDP showed the highest values of FC and EC, indicating its potential as an ingredient in sausages, bologna, soups, and cakes, whereas the good gel formation ability of ADP suggests its suitable application for puddings and jellies. All food formulations require distinct techno-functional properties, and all properties should be carefully considered to ensure their proper food application. Therefore, this study provides a clear resource on the appropriate selection of a defatting solvent for *G. bimaculatus* according to the final purpose of the food product.

## Figures and Tables

**Table 1:** Proximate analyses of *G. bimaculatus* and three defatted protein concentrates.

	Crude Protein (%, DM)	Crude Fat (%, DM)	Crude Ash (%, DM)	* Carbohydrate (%, DM)	Yield (%, DM)
FGP	51.3 ± 0.2 ^a^	17.3 ± 0.2 ^d^	4.52 ± 0.11 ^a^	26.9 ± 0.5 ^a^	-
EDP	62.5 ± 0.2 ^d^	0.73 ± 0.07 ^a^	4.76 ± 0.14 ^a^	32.1 ± 0.4 ^b^	77.7 ± 1.5 ^a^
HDP	60.7 ± 0.3 ^c^	1.83 ± 0.23 ^b^	5.21 ± 0.21 ^b^	32.3 ± 0.7 ^b^	79.3 ± 2.1 ^a^
ADP	60.2 ± 0.2 ^b^	2.56 ± 0.1 ^c^	5.27 ± 0.2 ^b^	32.0 ± 0.1 ^b^	83.5 ± 1.6 ^b^

FGP, freeze-dried *Gryllus bimaculatus* powder; EDP, ethanol-defatted protein concentrate; HDP, hexene-defatted protein concentrate; ADP, acetone-defatted protein concentrate; DM, dry matter. * Carbohydrate (%) = 100% − crude protein (%) − crude fat (%) − crude ash (%). Different letters in each column show a significant difference (*p* < 0.05) between means.

**Table 2:** Techno-functional properties of *G. bimaculatus* and three defatted protein concentrates.

Samples	WHC (g/g DM)	OHC (g/g DM)	Foaming Properties		EC (mL/g DM)	GF (%)
			FC (%)	FS (%)		
FGP	2.03 ± 0.19 ^a^	1.57 ± 0.02 ^a^	13.5 ± 1.1 ^a^	92.3 ± 0.9 ^c^	444 ± 24 ^a^	18.0 ± 0.0 ^c^
EDP	2.99 ± 0.30 ^b^	2.31 ± 0.07 ^c^	34.9 ± 1.1 ^b^	76.5 ± 1.0 ^a^	515 ± 10 ^b^	16.0 ± 0.0 ^b^
HDP	2.12 ± 0.15 ^a^	2.28 ± 0.02 ^bc^	61.1 ± 3.0 ^c^	73.9 ± 1.4 ^a^	558 ± 19 ^c^	16.0 ± 0.0 ^b^
ADP	2.33 ± 0.23 ^a^	2.02 ± 0.31 ^b^	39.7 ± 3.0 ^b^	82.4 ± 1.8 ^b^	538 ± 12 ^bc^	15.0 ± 0.0 ^a^

WHC, water holding capacity; OHC, oil holding capacity; FC, foaming capacity; FS, foaming stability; EC, emulsion capacity; GF, gel formation ability; FGP, freeze-dried *Gryllus bimaculatus* powder; EDP, ethanol-defatted protein concentrate; HDP, hexene-defatted protein concentrate; ADP, acetone-defatted protein concentrate; DM, dry matter. Different letters in each column show a significant difference (*p* < 0.05) between means.

**Table 3:** Protein secondary structural composition and surface hydrophobicity of soluble proteins from *G. bimaculatus* and three defatted protein concentrates.

Samples	Protein Secondary Structural Composition (%)				Protein Surface Hydrophobicity
	α-Helix	ß-Sheet	ß-Turn	Random Coil	
FGP	22.8 ± 0.8 ^b^	31.0 ± 1.4 ^b^	12.4 ± 0.8 ^a^	33.8 ± 2.3 ^a^	267.7 ± 20.8 ^a^
EDP	18.7 ± 0.8 ^a^	30.3 ± 1.2 ^b^	12.5 ± 1.0 ^a^	38.6 ± 1.7 ^b^	290.1 ± 15.9 ^b^
HDP	22.0 ± 1.5 ^b^	29.0 ± 1.0 ^b^	12.5 ± 1.1 ^a^	36.6 ± 2.7 ^ab^	341.3 ± 10.1 ^c^
ADP	22.1 ± 1.3 ^b^	26.3 ± 1.2 ^a^	12.0 ± 0.8 ^a^	39.6 ± 2.0 ^b^	325.0 ± 14.2 ^bc^

FGP, freeze-dried *Gryllus bimaculatus* powder; EDP, ethanol-defatted protein concentrate; HDP, hexene-defatted protein concentrate; ADP, acetone-defatted protein concentrate. Different letters in each column show a significant difference (*p* < 0.05) between means.

## Data Availability

The data presented in this study are available on request from the corresponding author.

## Sample Availability

Not available.
